# Clinical Guidelines in the Management of Frozen Shoulder: An Update!

**DOI:** 10.1007/s43465-021-00351-3

**Published:** 2021-02-01

**Authors:** Vivek Pandey, Sandesh Madi

**Affiliations:** Department of Orthopaedics, Kasturba Medical College, Manipal, Manipal Academy of Higher Education, Udupi, 576104 India

**Keywords:** Frozen shoulder, Adhesive capsulitis, Shoulder, Treatment, Conservative, Manipulation, Arthroscopic capsular release, Review

## Abstract

Among all the prevalent painful conditions of the shoulder, frozen shoulder remains one of the most debated and ill-understood conditions. It is a condition often associated with diabetes and thyroid dysfunction, and which should always be investigated in patients with a primary stiff shoulder. Though the duration of ‘traditional clinicopathological staging’ of frozen shoulder is not constant and varies with the intervention(s), the classification certainly helps the clinician in planning the treatment of frozen shoulder at various stages. Most patients respond very well to combination of conservative treatment resulting in gradual resolution of symptoms in 12–18 months. However, the most effective treatment in isolation is uncertain. Currently, resistant cases that do not respond to conservative treatment for 6–9 months could be offered surgical treatment as either arthroscopic capsular release or manipulation under anaesthesia. Though both invasive options are not clinically superior to another, but manipulation could result in unwarranted complications like fractures of humerus or rotator cuff tear.

## Introduction

Among almost all conditions of the shoulder, frozen shoulder (FS) has been most debated right from its terminology to the most optimal treatment and prognosis. Although the term FS and adhesive capsulitis have been used quite extensively, current ISAKOS guidelines favour use of the term *frozen shoulder* and discourage adhesive capsulitis as there are no adhesions in the shoulder joint [1]. The ISAKOS Upper Limb Committee has classified a stiff shoulder into the primary idiopathic stiff shoulder (frozen shoulder) and secondary stiff shoulder [1]. FS or primary idiopathic stiff shoulder terms are used to describe a stiff shoulder that develops without any specific trauma or any underlying disease process. The patient can have a condition that is known to have a link to stiffness (diabetes, thyroid disorders) but not necessarily known to cause stiffness. Secondary stiffness is reserved to describe shoulder stiffness with a known underlying cause such as trauma, infection, or inflammatory disorder. The prevalence of frozen shoulder is estimated to affect 2–5% of the population [2, 3], and affects men more than women [4]. The peak incidence is observed between 40 and 60 years [5]. 20% of patients develop similar symptoms in the opposite shoulder [4, 6]. Bilateral simultaneous involvement could be observed in 14% of the patients [7].

*Associated conditions:* Primary frozen shoulder is idiopathic, *but t*wo conditions are classically associated with FS; diabetes mellitus (DM) and thyroid dysfunction. The incidence of frozen shoulder in diabetic patients could vary from 10.8 to 30% [8, 9] with a tendency of more severe symptoms and resistance to treatment [10]. The prevalence of DM is ten times higher in patients with frozen shoulder, and higher HbA1C in a poorly controlled diabetes is associated with the development of FS [11]. Several studies have confirmed higher prevalence (27.2%) and incidence (10.9%) of hypothyroidism in patients with FS [12, 13]. Another recent study suggested 2.69 times higher risk of developing FS in patient with thyropathy [14]. Other associated conditions with FS are smoking, cardiac disease, Parkinson’s disease, stroke, neck and cardiac surgery, hyperlipidaemia and Dupuytren’s contracture [1, 15].

*Pathology*: FS is characterized by intense inflammatory changes in capsule indicating a role of inflammatory mediators (interleukins, cytokines, B- and T-lymphocytes, growth factors, matrix metalloproteinases, tumor necrosis factors and fibroblast activation markers) and disturbance in local collagen translation, which result in global fibroplasia [16–19]. Macroscopically*,* the capsule of the FS appears thick, congested and inflamed, particularly around the rotator interval and anteroinferior capsule along with thickened coracohumeral ligament (CHL) and superior–middle–inferior glenohumeral ligaments resulting in loss of flexion, abduction and rotations [20, 21]. The tissue samples from FS reveal dense collagen matrix and high population of fibroblasts and contractile myofibroblasts, a process similar to Dupuytren's contracture, with the fibrotic process predominantly limited to anterior capsule [22, 23]. An early immune response with elevated levels of alarmins, binding to the receptor of advance glycation end products and accrued irreversible crosslinks between various collagen protein molecules through glycosylation is observed at the beginning of the cascade [24, 25]. Increased expression of vascular endothelial growth factors (especially in diabetics with high glycosylated haemoglobin), nerve growth factor receptor and neoangiogenesis are also noted, and that may help explain severe pain and stiffness in patients with FS [26, 27]. In summary, the frozen shoulder appears to start as an inflammatory reaction in capsule with associated synovitis that progresses to the fibrotic contracture of the capsule.

## Clinical Features and Clinicopathological Stages

Patients with FS complain of insidious onset of pain and stiffness without any preceding traumatic, infective, or inflammatory event. Pain is usually poorly localized, around the shoulder and is almost always troublesome in the night while the patient lies on the affected side. The index shoulder examination reveals global restriction of both active and passive range of movements (ROM) at least in two planes, and that is one of the critical findings. The loss of external rotation with arm by the side of the chest is one of the earliest signs. According to ISAKOS guidelines; if the range of movement is less than 100° in forward flexion, less than 10° in external rotation, and less than L5 vertebral level in internal rotation, it is defined as a global restriction of ROM [1]. In a recently published FROST trial, investigators defined FS as a condition characterised by the restriction of passive external rotation in the affected shoulder to less than 50% of the opposite shoulder with normal radiographs [28]. The strength of the rotator cuff is relatively unaffected.

Traditionally, FS is described in ‘three clinico-pathological stages’ (freezing, frozen, and thawing), which we find practical to understand and explain to the patients and decide the treatment plan [29–31]. Another classification suggested by Hanchard et al. as ‘pain predominant’ and ‘stiffness predominant’ is also useful in clinical practice [32].

The principal clinical characteristics of three stages are pain, pain and stiffness, and stiffness, respectively.

- Freezing stage (stage 1): It may last for 2–6 months. Clinically, stage 1 is predominantly characterized by moderate-severe pain and partial restriction of ROM. Pathologically, it is characterized by a slow onset of widespread inflammation involving capsule and synovium of the shoulder joint resulting in gradual onset of 'pain' as a principal symptom. Mere pain and only terminal loss of ROM in the early period of freezing stage of FS could be confused with rotator cuff tendinopathy as latter also presents with painful loss of terminal ROM. However, ROM does not progressively worsen in RC tendinopathy whereas it continues to worsen at every follow up in FS.
- Frozen stage (stage 2): It may last for 4–12 months. Clinically, this stage is characterized by both ‘pain and stiffness’ in varying proportions. Patients in the early phase of stage 2 have more pain while later phase of stage 2 comprises of more stiffness than pain. Pathologically, it is characterized by a gradual diminution in inflammation and onset of widespread fibrosis of capsule and ligaments which results in gross restriction of ROM.
- Thawing stage (stage 3): It may last for 6–26 months. Clinically, this stage is characterized by minimal pain and gradual resolution of stiffness. Pathologically, it is characterized by gradual resolution of inflammation and fibrosis, resulting in minimal pain and the progressive return of movements.

## Investigations

The relationship between an established DM and thyroid dysfunction with FS is a known fact but the evidence to investigate an apparently ‘normoglycemic FS patient’ with fasting blood glucose level and haemoglobin A1C is mounting as several studies have confirmed that latter two investigations are often deranged in patients with FS [11, 33–35]. Further, thyroid dysfunction should also be investigated if there is a clinical suspicion [12]. Primary frozen shoulder is essentially a clinical diagnosis, and, therefore, the radiographic studies are performed to exclude other secondary causes of shoulder pain such as calcific tendinitis, rotator cuff tear, arthritis of glenohumeral and acromioclavicular joint or a neoplastic process. The plain radiograph of the shoulder is essentially normal in patients with 1° FS. However, osteopenia of the humeral head is not uncommon and should alert the surgeon if manipulation is planned [36]. MRI is not routinely performed in patients with FS to diagnose the condition. However, it could be done to rule out any secondary cause of FS if there is a clinical suspicion. In an early freezing stage, MRI may show edema of joint capsule and obliteration of the sub-coracoid fat triangle. In the frozen stage; MRI shows capsular and CHL thickening, poor capsular distension, volume reduction of the axillary pouch, and scar formation in the rotator interval [37]. Recently, ultrasonography (USG) has emerged as a primary diagnostic tool in establishing the diagnosis of the frozen shoulder which reveals thickened CHL (mean thickness 1.2 mm, observed in 96.7% cases), increased vascularity in the rotator interval, and presence of hypoechoic soft tissue thickness in the rotator interval [38].

## Treatment

By and large, conservative treatment of frozen shoulder is successful in up to 90% patients [39–41]. Only a few require operative intervention in the form of manipulation under anaesthesia (MUA) or arthroscopic capsular release (ACR). In clinical practice, the strategy opted to treat frozen shoulder often depends upon the clinicopathological stage of frozen shoulder. Further, the patients with FS should also be treated for underlying medical disorder (DM and thyroid dysfunction) as poor control of these disorders may result in prolonged severity of disease. Although diabetics often tend to have severe disease and require prolonged conservative treatment and require surgical treatment more often than non-diabetics [42–44], one must not give up conservative treatment trial and expedite surgical treatment in diabetics.

- Freezing stage: Since this stage is characterized by the presence of intense pain due to underlying capsule–ligament–synovium inflammation, the treatment strategy deployed in this stage should aim at minimizing pain. Many options are used to alleviate pain, such as NSAIDs, steroids (local or oral), and pain-relieving physiotherapy (PT). A point needs to be stressed that none of these measures would relieve the pain entirely, and the entire exercise of pain minimization is aimed at enabling the patient to perform activities of daily living (ADL) with more ease, sleep better and gradually initiate the joint mobilisation PT. The mobilisation PT is principally aimed at ‘retaining’, and ‘slowly regaining’ the ROM. Of note-during the stage of intense pain, vigorous mobilization PT must be avoided as it can exacerbate the inflammation resulting in increased intensity of pain.
- Frozen stage: In this stage; pain is less, but the loss of ROM is profound due to fibrosis of capsulo-ligament complex. Hence the treatment strategy should be principally aimed to gradually ‘increase and regain’ the ROM by deploying a structured and well-sustained mobilisation PT program. Hydrodilatation (HD) could also be used as an adjunct in early frozen stage to break capsular fibrosis and accelerate the gain in ROM. Occasional analgesics keep the pain at bay. If sincere attempts of PT for several weeks–months and or HD fail to improve functional ROM and pain, MUA or ACR could be considered to accelerate the functional recovery in terms of regaining ROM and minimizing pain.
- Thawing stage: This stage is characterised by minimal or no pain and gradually improving ROM for past several weeks. Hence, sustained PT remains the mainstay of the treatment in this stage, which aims to gradually regain the ‘functional’ followed by total recovery of shoulder ROM. Any surgical interventions are hardly required in this stage.

### Non-operative Treatment of Frozen Shoulder

1. NSAIDs and other analgesics: NSAIDs remain one of the most common medical intervention in treating frozen shoulder [45]. A short course of NSAIDs for 2–3 weeks is very frequently used to minimise intense pain of the freezing stage. However, course of NSAIDs does not alter course of the frozen shoulder but enables the patient to carry out their ADLs in a more relaxed fashion and perform PT (retaining ROM) with ease. However, there is a paucity of high-quality studies discussing the utility of NSAIDs in comparison to other drugs, especially corticosteroids. In patients with NSAID allergy or contraindication, Opioid analgesics can be used.
2. Corticosteroid: Apart from NSAIDs, steroids are the second most commonly used drugs in the treatment of the frozen shoulder. Both oral steroid and local steroid injections are widely used. A paramount point to note that steroids in any form are beneficial only in early stages (freezing and early frozen) of frozen shoulder to control inflammation and ensuing pain, and there may not be any rationale to prescribe it in late stages of frozen shoulder with established fibrosis without much inflammation.
3. Oral steroids: In several high-quality studies, moderate evidence was found in favour of oral steroid for improving pain, ROM and function when prescribed for ‘short term’ (6 weeks) in stage 1 [46, 47]. However, the effects were not maintained beyond 6 weeks after stopping it. Nevertheless, disastrous complication of avascular necrosis of femoral head has to be feared of, even with a short course of oral steroid [48].
4. Local injectable steroids: Local injectable steroid is most frequently deployed medical method to provide relief from severe pain in freezing stage of FS. Systematic reviews and metanalysis have confirmed strong evidence in favour of steroid injections in improving pain and ROM as compared to placebo in the short term, and moderate evidence in the midterm [46, 49]. Two RCTs concluded that injectable steroid provide superior clinical results compared to oral steroid [50, 51]. Steroid injection is certainly superior to PT in reducing pain but evidence is conflicting regarding restoration of ROM while comparing steroid injection with PT or MUA [46].
  Furthermore, many issues regarding use of local injectable steroids such as optimal dose [52, 53], single or multiple injection, site of injection (intraarticular/subacromial/rotator interval) [54, 55], molecule (Triamcinolone/Methylprednisolone) [56–58], injection with or without imaging [59] remain contentious and are briefly mentioned in Table 1.
  Of-note, steroid injections carry a risk of a transient increase in blood glucose levels (BGLs) occurring within 1–5 days in diabetic patients [60]. However, the rise in BGLs returns to baseline within 24 h to 10 days, and the benefit of steroid injection in improving pain scores and function outweighs any transient increase in BGLs. Nevertheless, steroid injections must be avoided in patients with uncontrolled Diabetes, especially if BGL is more than 250 mg% [60].
  *Adverse events with injectable steroids:* Minor complications such as facial flushing, chest or shoulder pain, dizziness and nausea are reported due to vasovagal reactions during injection [61]. Furthermore, Triamcinolone injections must be avoided in patients with retroviral therapy due to the risk of drug interaction causing iatrogenic Cushing syndrome [62].
5. Physiotherapy (PT): Along with NSAIDs and steroids, PT remains one of the cornerstones in the treatment of the frozen shoulder. The arms of PT consist of ‘pain-relieving PT’, ‘mobilization PT’ and ‘strengthening PT’. In the freezing stage, it is better to use pain-relieving PT and avoid aggressive mobilization techniques as latter can exacerbate the pain. There are various modalities of ‘pain relieving PT’ such as Laser, short wave diathermy, ultrasound and hot packs [46, 63]. PT, along with NSAIDs or steroid injection, is better in providing symptomatic relief than PT alone [64–66].
  Once pain decreases, ‘mobilization PT’ can be started to retain and gradually regain ROM. The patients receiving PT must start with 3–4 sessions per day, with each session of 10–15 min, comprising of active-assisted ROM exercises, including forward elevation, abduction, rotations, and cross-body adduction. This must be combined with scapular and cuff rehabilitation along with core strengthening. Grigg’s et al. confirmed that patients in phase II of frozen shoulder report high satisfaction with four-direction stretching exercise [67]. In the late frozen stage, low-and high-grade mobilization techniques could be implemented to regain the ROM along with ‘muscle strengthening PT’ [46].
  In comparison to PT vs. MUA in the frozen stage, a high-quality RCT confirmed the superiority of MUA compared to home exercise alone [68].
6. Hydrodilatation (HD): In late freezing or early frozen stage, HD of the glenohumeral joint using saline, steroid, local anaesthetic agent is supposed to distend the capsule by breaking the ‘early intracapsular fibrosis’ which helps in improving ROM [2]. A single HD procedure is superior to placebo in improving ROM, pain, and function in the short term [69]. However, more than one repeated HD after 2 weeks has no added effect over a single HD procedure [70]. Nevertheless, HD may not offer any advantage in comparison to IA steroid injection [71, 72].
7. Calcitonin: Calcitonin decreases systemic inflammatory response and stimulate the release of endorphins [73]. Yang et al. confirmed that addition of salmon calcitonin in biopsied tissues from frozen shoulder improves mRNA expression of fibrosis-related molecules and decreased the enhanced cell-substrate adhesion ability of frozen shoulder [74]. A level II RCT concluded that the addition of Calcitonin along with PT and NSAIDs alleviates pain and functional outcome better than mere PT and NSAIDs [73]. However, further research is required in this area to validate the role of Calcitonin in frozen shoulder.
8. Extracorporeal shock wave therapy (ECSWT): An RCT involving 40 patients treated with ECSWT versus oral steroid confirmed that ECSWT significantly improves the functional outcome and ROM without any adverse events [75]. In a systematic review of 19 trials (1249 patients), the use of ECSWT did not beget any major adverse event [63]. Further, ECSWT is a suitable alternative in patients with uncontrolled diabetics or where oral steroids cannot be prescribed.
9. Acupuncture: Though few centres have tried using acupuncture in the treatment of FS and reported reasonable relief in pain and improved forward flexion [76, 77], there is little evidence in literature for its routine use in the treatment of primary FS [78].
10. Nerve block: Several authors report that single or multiple injections to block Suprascapular nerve in the treatment of frozen shoulder result in improved pain score and ROM [79, 80]. However, there is lack of high-quality evidence in favour of the nerve block and is not routinely performed.

**Table 1:** Summary of various debatable parameters regarding injectable steroids

	Parameters	Reference	Conclusions
1	High dose (40 mg), low dose (20 mg) or very low dose (10 mg) steroid	Kim et al., RCT, 2018 [52] Yoon et al., RCT, 2013 [53]	1. No difference between 40 mg vs 20 mg 2. 10 mg is less effective than 40 mg
2	Single vs. Multiple injections	Erickson et al., 2019 [44]; retrospective study of 1377 patient	Multiple are no better than single injection in improving clinical outcome
3	Site: IA vs SA vs RI	Shang et al., Meta-analysis, systematic review, 2019 [54]	1. No overall significant difference 2. Pain scores better in IA groups 3. IR better in SA groups 4. SA injection result in lesser BGL fluctuation
		Sun et al., RCT, 2018 [55]	Single injection into SA, IA and RI resulted in better pain, ROM and functional scores in RI group
4	Triamcinolone (TA) vs. Methylprednisolone (MTP)	Sakeni et al., Level II, 2007 [57] One injection a week for 3 weeks	TA gave superior result in resistant cases and Diabetics compared to MTP
		Choudhary et al., 2015 [56]; Three injection every three weeks in either group	TA group had better pain scores and ROM
		Lopez et al., 2008 [58]	More relief of pain in MTP than TA
5	With or without image (USG or fluoroscopic) guidance	Song et al., Systematic review, 2014 [59]	Added benefit of Image guided injections over blind injection in improving pain and ROM. However, needs further evaluation

*IA* Intraarticular, *SA* Subacromial, *RI* Rotator interval IR, Internal rotation, *ROM* range of movement, *USG* Ultrasonography, *BGL* blood glucose level. Number in [] denotes reference in the text

### Operative Management of Frozen Shoulder

Invasive operative methods (manipulation or surgical release of capsule) to improve function in patients with primary FS are recommended only when an extended conservative treatment for a period of 6–9 months fails to provide significant relief to the patient [35, 41, 81]. The surgical techniques consist of manipulation under anaesthesia (MUA) and arthroscopic capsular release (ACR).

**Fig. 1:**
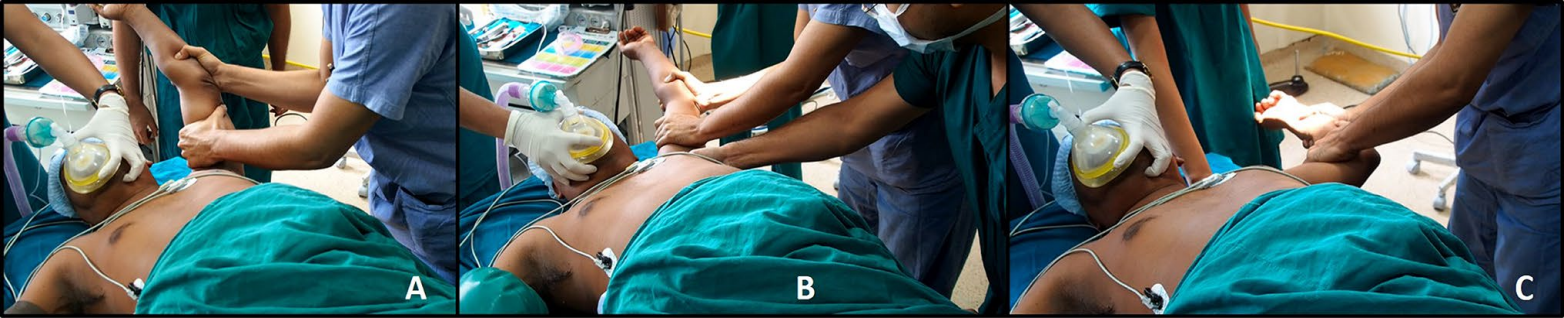
**a**–**c** Shows the MUA of left shoulder with a ‘short-lever arm’ while arm being taken in flexion, abduction and external rotation in 90^°^ abduction. Of-note: during abduction beyond 90^°^, head of the humerus is supported with a fist of assistant in axilla to prevent inferior subluxation of head while tearing of inferior capsule. During external rotation movement in 90^°^ abduction, the scapula is stabilised by the assistant’s hand over the scapula

**Fig. 2:**
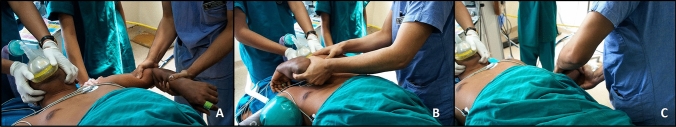
** a**–**c** Shows MUA of left shoulder with a ‘short-lever arm’ while arm is taken in internal rotation in 90^°^ abduction, cross-chest adduction and external rotation with arm by the side of chest. During internal rotation movement in 90^°^ abduction, the scapula is stabilised by the assistant’s hand over the scapula

1. Manipulation under anaesthesia: MUA is a method wherein fibrosed capsulo-ligament complex of shoulder, which is a hindrance in regaining ROM, is forcibly broken by manoeuvring the shoulder across the ROM under anaesthesia. Krall et al. suggested that MUA is an effective method to improve function in patient with refractory FS in stage II, external rotation < 50% compared to opposite side and failure to respond to IA steroid infiltration [82]. Of note: MUA should not be performed for secondary stiffness of the shoulder, and such patients must undergo arthroscopic capsular release if need be.
  *Technique*: Under anaesthesia, the arm of the patient is held with a short lever and shoulder is gently moved in flexion, abduction followed by external and internal rotation in 90° abduction (Figs.1 and 2). Next, the shoulder joint is taken into external rotation with arm by the chest followed by cross-chest adduction. These manoeuvres result in tearing of fibrosed capsule and ligament, which can often be felt or heard during MUA. Of note- no movement should be forced if ‘excess’ resistance is felt during the range of that movement, and it is better to perform the next manoeuvre rather than applying too much force to regain that movement to avoid complications. At the end, all movements are repeated to confirm that end range has been achieved. The authors prefer to inject 40 mg of triamcinolone and 10 ml of 0.25% Bupivacaine to minimise postoperative pain and inflammation. Many other authors too prefer post MUA injection of steroid and local anaesthetic agent [83, 84]. However, concrete evidence for the benefit of the same is lacking.
  Many studies have reported good to excellent long-term clinical outcome after MUA [85–87]. Said that, several debatable pertinent questions regarding MUA; such as timing [88], with or without steroid injection [84], its efficacy in comparison to other conservative options [68, 89], and role in diabetics [42, 90–92] are mentioned in Table 2.
  Complications of MUA: Literature reports an overall complication rate of 0.4%, and a re-intervention rate of 14% [82]. Although MUA improves flexion and abduction, limitation in rotation in early phase after MUA remains a concern as surgeons often avoid forcible rotations during MUA due to the fear of complications. Albeit rare, various complications can occur during MUA, especially while achieving a terminal range of movement such as Humerus shaft fracture, rotator cuff tear, shoulder dislocation, labral tear, nerve injury, and complex regional pain syndrome [90, 93–96].
2. Arthroscopic capsular release: Although all high-quality clinical studies have failed to reveal any major advantage of ACR over MUA [28, 97]; of late, ACR has emerged as ‘preferred’ surgical option for the treatment of refractory FS as ACR allows controlled and precise release of fibrosed capsule–ligament complex under vision avoiding the said complications of MUA under the same anaesthetic burden [41, 95]. Further, ACR enables the surgeon to thoroughly inspect and treat a “clinically or radiologically missed” concomitant lesion of the cartilage, rotator cuff, labrum and subacromial space, if any, which may be contributing to the pathology. Like MUA, ACR should be performed between 6 and 8 months of onset of frozen shoulder.

**Table 2:** Summary of various contentious parameters regarding manipulation under anaesthesia (MUA) such as timing, with or without steroid injection, comparison with other conservative method, comparing two commonly used steroid molecules and outcome in diabetic vs. noon-diabetic frozen shoulders

	Parameters	Reference	Conclusions
1	Timing of MUA (early or delayed)	Vastamaki et al., 2015 [88]	Delayed between 6 and 8 months while shoulder is in late frozen phase. Early MUA in freezing or early frozen phase could result in aggravation of symptoms
2	With or without intraarticular steroid injection (after MUA, in operating room itself)	Kivimaki et al., RCT, 2001 [84]	No difference. Hence, authors recommended that addition of steroid is of no use
3	Comparison with other conservative methods such as therapeutic exercise; steroids and distention	Kivimaki et al., RCT, 2007 [68] Jacobs LG et al., RCT, 2009 [89]	No difference
4	Outcome of MUA in diabetics vs controls	Hamdan et al., 2003 [90]	Diabetics have poor outcome
		Wang JP et al., 2010 [42]	No difference
		Jenkins et al., 2012 [91]	36% of diabetics may require repeat MUA compared to 15% controls
		Woods et al., 2017 [92]	38% risk of repeat MUA in diabetics compared to 18% as a group

*RCT* randomised controlled trial. Number in [] denotes reference in the text

Many studies have shown excellent short-, mid- and long-term results both in terms of lasting pain relief and ROM gains with ACR [98–101]. Comparing diabetics with non-diabetics, a recent systemic review concluded that clinical outcomes after ACR were inferior (more residual pain, reduced motion) in a diabetic patient compared to non-diabetic, and that must be explained to the patient during pre-ACR counselling [102].

**Fig. 3:**
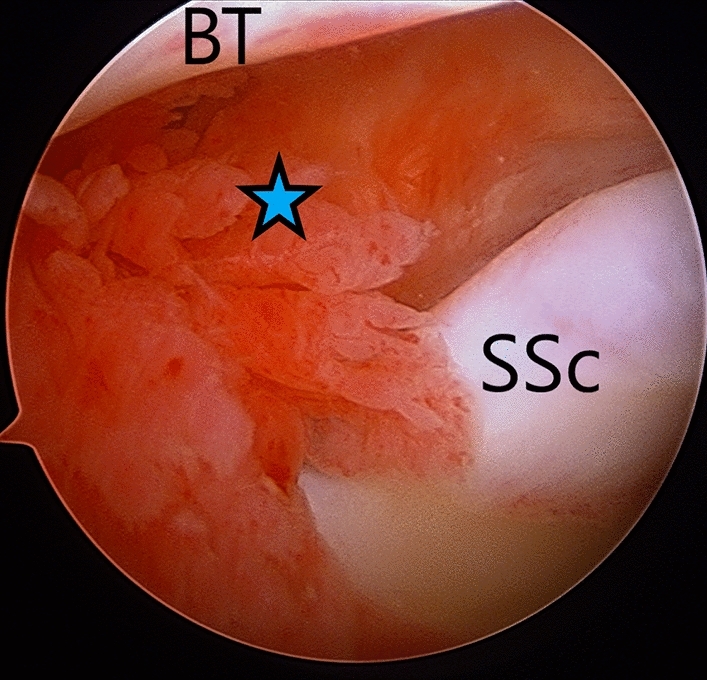
Arthroscopic view (from posterior portal) of inflammed and contracted rotator interval (blue star) of right-side frozen shoulder. *SSc* subscapularis, *BT* biceps tendon

**Fig. 4:**
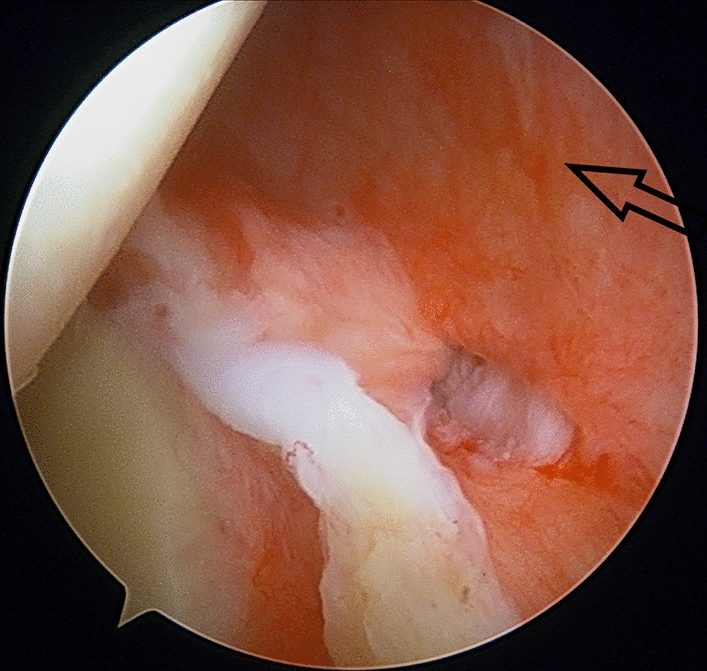
Arthroscopic view (from anterior portal) of inflamed synovium-capsule over the infraspinatus

*Technique*: Under anaesthesia, diagnostic arthroscopy is performed from the posterior portal. The entry into the joint is often tricky, and care must be taken while inserting the trocar to avoid damage to the articular cartilage of humerus or glenoid. In case entry in the joint is not possible, force must be avoided, and gentle, controlled manipulation of joint should be done to break the extremely tight capsule, and that would enable the surgeon to insert the arthroscope. Lafosse et al. recommended lateral entry via rotator interval in tight shoulders to avoid damage to intraarticular structures during forcible entry [100]. In almost all cases; the rotator interval is contracted and inflamed (Fig. 3), intra-articular part of the biceps tendon may reveal inflammation, and synovitis is often present in the joint, especially over the capsule covering the under-surface of the supra-and infraspinatus (Fig. 4). Through the standard anterior portal, the RI and CHL are released using a radiofrequency device (RFD), and synovitis is gently debrided. The tight MGHL is released followed by release of anterior capsule till the anteroinferior corner of the capsule using RFD. The scope is shifted to the anterior portal, and the posterior capsule is released till the posteroinferior corner. Due to the proximity of the inferior capsule to the axillary nerve, the inferior-most capsule is not released surgically but is broken with gentle MUA at the end of the procedure [103]. Literature remains contentious regarding clinical outcome after limited anterior capsule release and MUA vs. circumferential release [41]. Next, arthroscope is shifted to the subacromial space, and subacromial adhesion or inflamed bursa, if any, is debrided. With arthroscope in lateral portal, CHL is again inspected, and should be released if found to be incompletely released. Further, adhesions over the bursal and articular side of subscapularis muscle is released up to the base of the coracoid to improve the mobility of the subscapularis, and thereby improving external rotation. At the end of the procedure, surgeon must gently move the shoulder in all directions to ensure that the entire fibrotic capsule–ligamentous complex is released [41]. Post MUA or ACR, authors prefer to inject 40 mg of Triamcinolone along with 10 ml of 0.25% Bupivacaine to minimise post-procedure inflammation and pain. Although many authors prefer injecting steroid post-ACR [83, 104–106], only a few report superior outcome after the injection [106]. However, larger consensus regarding utility of steroid injection post-ACR is lacking.

*Pain control and rehabilitation after MUA and ACR:* Adequate pain control for 2–3 weeks using NSAIDs and local ice pack is quintessential for pain relief. Structured PT must follow immediately after the procedure, and continue for 4–6 months aiming to retain 'regained intra-operative' ROM. The PT program should consist of early passive and active-assisted ROM along with scapula stabilisation followed by active ROM combined with strengthening exercises for rotator cuff, scapular muscles, and core rehabilitation.

MUA vs. ACR: Despite all said advantages of ACR over MUA, literature has failed to prove clinical superiority of ACR over MUA [28, 83, 97]. A recently published triple arm pragmatic superiority randomised controlled trial by Rangan et al. concluded that PT, MUA and ACR are not superior to each other in treatment of resistant FS [28]. Further, Rangan et al. concluded that ACR is more costly and associated with serious adverse events (4%) while MUA is most cost-effective procedure [28]. However, in a systematic review of 22 studies (21 were level IV), Grant et al. concluded that rate of complications with either procedure (MUA and ACR) is less than 0.5% [97].

## Conclusion

While managing FS, clinician must investigate and manage the patient for any associated conditions especially diabetes and thyroid dysfunction alongside treating the FS. With the availability of trained musculoskeletal sonologist along with advanced sonographic machines, USG could be considered as a primary tool to confirm the diagnosis of FS and rule out secondary disorders rather than straightaway subjecting the patient to MRI. Largely, combination of conservative treatment works quite well in most patients of FS with good to excellent outcomes and must be tried for at least 6–9 months before embarking upon any invasive procedure. Nevertheless, no single conservative treatment option is found to be remarkably superior to others, and multimodal treatment comprising NSAIDs, steroid, and structured-sustained PT remain all-season favourites. Among the invasive procedures; both MUA and ACR seem to be equivocal in achieving functional improvement, but the latter is currently more preferred as it is largely devoid of most complications listed for MUA. Early and sustained PT along with good analgesia is quintessential postoperatively for a complete recovery. Considering overall recovery and achieving satisfactory functional outcomes, Diabetic patients continue to fare poorly as compared to non-diabetics.
